# Full-length transcriptomes of 25 grassland plant species

**DOI:** 10.1038/s41597-025-05280-6

**Published:** 2025-06-02

**Authors:** Chongyi Jiang, Zixia Huang, Cynthia Meizoso, Gaby Kumpfmüller, Jochen B. W. Wolf, Holger Schielzeth

**Affiliations:** 1 Population Ecology Group, Institute of Ecology and Evolution, Friedrich Schiller University, Jena, Germany; 2 School of Biology and Environmental Science, University College Dublin, Dublin, Ireland; 3 Division of Evolutionary Biology, Faculty of Biology, LMU Munich, Grosshaderner Str. 2, 82152 Planegg-Martinsried, Germany

**Keywords:** Grassland ecology, Plant genetics

## Abstract

Grasslands are essential, biodiverse ecosystems providing critical ecosystem services. Despite their ecological and economic value, transcriptomic resources for wild grassland species to support eco-evolutionary and functional genomic studies remain limited. Here, we present full-length transcriptomes for shoot tissue from 25 wild grassland plant species collected from a long-term biodiversity experiment (the Jena Experiment). Using PacBio Iso-Seq technology, we generated a total of 522.45 million subreads, which were assembled into unique transcripts for each species independently. This resulted in an average of 49,180 transcripts per species, of which 68.6% were successfully annotated using the Swiss-Prot database. Furthermore, 40.3% of the transcripts contained complete open reading frames (ORFs), while 31.4% had incomplete ORFs. More than 36.8% of the transcripts were identified as non-coding RNAs. On average, 5.08% of the bases across all transcriptomes were flagged as repetitive elements. This dataset offers a valuable full-length transcriptomic resource for studying gene expression, alternative splicing, and evolutionary patterns in grassland species, paving the way for future research in functional genomics and conservation.

## Background & Summary

Grassland ecosystems are among the most biodiverse biomes on Earth^1–3^, playing crucial roles in carbon sequestration^4,5^, soil fertility^6,7^, and supporting wildlife^8–10^. However, grassland ecosystems are increasingly threatened by climate change, land transformation, and habitat fragmentation^11–13^. Functional genomic research in grassland plants enables a deeper understanding of how these ecosystems respond to environmental changes^14–16^ and supports the development of effective conservation strategies.

Next-generation sequencing (NGS) technologies have revolutionized biological research by enabling rapid and cost-effective genome and transcriptome analysis^17–21^. Among these advances, Isoform sequencing (Iso-Seq) — a long-read approach based on single-molecule real-time (SMRT) sequencing developed by Pacific Biosciences — allows the capture of full-length transcripts^22,23^, facilitating detailed characterization of isoforms, alternative splicing events, and complete gene models^23–26^. These technologies have been widely applied in plant research^27^, particularly for economically important food crops^28–30^, where they have significantly advanced our understanding of the genetic foundations of domestication and disease resistance^30–32^. However, genomic and transcriptomic studies on non-model species, especially wild grassland plants, remain limited. Comprehensive transcriptomic data for these species are critically needed to improve our understanding of gene function, adaptation, and resilience in grassland ecosystems.

Here, we present the transcriptome inventory of 25 wild grassland plant species spanning a large phylogenetic range, generated using PacBio Iso-Seq technology (Table 1). These 25 species are dominant taxa in Central European grasslands, particularly within the long-term Jena Experiment, for which extensive ecological data have been accumulated^33–37^. We generated a total of 522.45 million subreads, which were assembled and subsequently annotated using the Swiss-Prot database. Furthermore, we identified non-coding RNAs (ncRNAs), coding sequences and transposable elements in all species. Our dataset represents the first large-scale collection of full-length transcripts for wild grassland species, providing invaluable insights into gene structure, isoform diversity, and evolutionary relationships in these ecologically significant organisms.

**Table 1:** Target species.

Species	Order	Family	Abbreviation	Functional group
*Ajuga reptans*	Lamiales	Lamiaceae	AjuRep	Small herb
*Alopecurus pratensis*	Poales	Poaceae	AloPra	Grass
*Arrhenatherum elatius*	Poales	Poaceae	ArrEla	Grass
*Bellis perennis*	Asterales	Asteraceae	BelPer	Small herb
*Crepis biennis*	Asterales	Asteraceae	CreBie	Tall herb
*Daucus carota*	Apiales	Apiaceae	DauCar	Tall herb
*Galium mollugo agg*.	Gentianales	Rubiaceae	GalMol	Tall herb
*Geranium pratense*	Geraniales	Geraniaceae	GerPra	Tall herb
*Holcus lanatus*	Poales	Poaceae	HolLan	Grass
*Knautia arvensis*	Dipsacales	Caprifoliaceae	KnaArv	Tall herb
*Lathyrus pratensis*	Fabales	Fabaceae	LatPra	Legume
*Leucanthemum vulgare agg*.	Asterales	Asteraceae	LeuVul	Tall herb
*Lotus corniculatus*	Fabales	Fabaceae	LotCor	Legume
*Luzula campestris*	Poales	Juncaceae	LuzCam	Grass
*Medicago* x *varia*	Fabales	Fabaceae	MedVar	Legume
*Plantago lanceolata*	Lamiales	Plantaginaceae	PlaLan	Small herb
*Plantago media*	Lamiales	Plantaginaceae	PlaMed	Small herb
*Primula veris*	Ericales	Primulaceae	PriVer	Small herb
*Prunella vulgaris*	Lamiales	Lamiaceae	PruVul	Small herb
*Ranunculus acris*	Ranunculales	Ranunculaceae	RanAcr	Tall herb
*Trifolium dubium*	Fabales	Fabaceae	TriDub	Legume
*Trifolium pratense*	Fabales	Fabaceae	TriPra	Legume
*Trisetum flavescens*	Poales	Poaceae	TriFla	Grass
*Veronica chamaedrys*	Lamiales	Plantaginaceae	VerCha	Small herb
*Vicia cracca*	Fabales	Fabaceae	VicCra	Legume

## Methods

### Sample collection and RNA preparation

Plant samples were collected from the Jena Experiment^38^, a long-term grassland biodiversity field site. In this study, shoot tissues from 25 selected grassland species were harvested for RNA extraction and sequencing (Table 1). Approximately 10 mg of tissue from each sample was homogenised with a −80 °C cold QIAGEN Tissuelyser II. Total RNA from species *Lathyrus pratensis*, *Trifolium pratense*, *Daucus carota*, *Primula veris*, *Luzula campestris*, and *Lotus corniculatus* was extracted using TRIzol reagent (Zymo Research, Direct-zol RNA Microprep Kits #R2062) following the manufacturer’s protocol. For the remaining species, RNA was extracted using a lysis buffer with β-mercaptoethanol (Roboklon, Universal RNA Purification Kit #E3598). For all samples, a cleanup step was performed with the OneStep PCR Inhibitor Removal Kit (Zymo Research). RNA purity and concentration were assessed using a NanoDrop ND-1000 spectrophotometer and Qubit^TM^ (Thermo Fisher Scientific). RNA integrity was confirmed by running samples on a 1.2% agarose.

### PacBio library construction and sequencing

PacBio Iso-Seq libraries were constructed following the standard Iso-Seq library preparation protocol provided by Pacific Biosciences. The process involved synthesizing full-length cDNA using the SMARTer® PCR cDNA Synthesis Kit (Takara Bio), followed by PCR amplification and purification. The synthesized cDNA was then subjected to damage repair and terminal repair. SMRTbell adapters were ligated to the ends of the double-stranded cDNA molecules. The Sequel Binding and Internal Control Kit 3.0 (Pacific Biosciences) was used to bind primers and polymerase to the library for sequencing. Purification of the final library was performed using AMPure PB magnetic beads (Pacific Biosciences), and the libraries were loaded onto the PacBio Sequel II system for sequencing. The raw sequencing data ranged from 13.64 Gbp to 31.67 Gbp per species, corresponding to between 15,119,410 and 26,635,333 subreads (Table 2). Variation in read yields was primarily due to differences in RNA extraction efficiency and may have affected transcriptome completeness.

**Table 2:** Summary of the transcriptome statistics.

Species	Subreads	ccs	Multiplets	Singletons	Total transcripts	Annotated by SwissProt	SwissProt sequences
*Ajuga reptans*	26,635,333	255,943	15,820	53,304	69,124	67.20%	12,803
*Alopecurus pratensis*	20,844,493	183,178	9,561	34,069	43,630	66.80%	8,083
*Arrhenatherum elatius*	23,139,526	242,412	15,816	47,826	63,642	65.40%	9,053
*Bellis perennis*	18,693,146	191,718	14,668	43,565	58,233	70.70%	11,331
*Crepis biennis*	18,494,462	156,075	9,886	36,963	46,849	67.10%	8,639
*Daucus carota*	18,860,036	156,012	10,794	29,142	39,936	75.70%	8,740
*Galium mollugo agg*.	21,007,128	202,396	15,644	39,741	55,385	71.90%	9,765
*Geranium pratense*	15,119,410	100,873	3,521	12,835	16,356	74.40%	5,588
*Holcus lanatus*	20,108,204	207,001	15,095	41,129	56,224	65.80%	8,653
*Knautia arvensis*	21,801,584	216,233	10,019	32,613	42,632	72.60%	8,658
*Lathyrus pratensis*	23,987,999	233,374	16,134	47,408	63,542	66.80%	10,272
*Leucanthemum vulgare agg*.	15,286,148	147,510	5,929	24,450	30,379	65.20%	7,602
*Lotus corniculatus*	17,258,842	144,178	12,668	42,599	55,267	72.00%	9,854
*Luzula campestris*	22,372,799	192,184	14,469	37,480	51,949	65.90%	9,817
*Medicago* x *varia*	21,844,887	199,338	13,819	43,211	57,030	70.80%	9,722
*Plantago lanceolata*	22,045,801	220,458	13,561	34,372	47,933	71.80%	9,363
*Plantago media*	26,081,676	253,971	18,589	51,626	70,215	70.40%	11,074
*Primula veris*	19,268,746	182,154	13,476	33,887	47,363	75.60%	9,744
*Prunella vulgaris*	18,920,021	176,694	13,963	37,621	51,584	73.20%	9,927
*Ranunculus acris*	24,712,278	229,973	12,664	39,861	52,525	63.20%	9,837
*Trifolium dubium*	23,470,386	245,758	16,473	46,324	62,797	73.20%	10,141
*Trifolium pratense*	18,955,447	146,947	6,333	20,351	26,684	71.10%	6,628
*Trisetum flavescens*	16,512,697	113,404	6,841	24,697	31,538	55.50%	6,061
*Veronica chamaedrys*	22,735,906	214,735	12,660	37,938	50,598	70.50%	9,560
*Vicia cracca*	24,295,044	165,099	7,937	30,137	38,074	53.20%	7,278
**Average**	**20,898,080**	**191,105**	**12,254**	**36,926**	**49,180**	**68.60%**	**9,128**

### Pacbio Iso-seq data processing

**Fig. 1:**
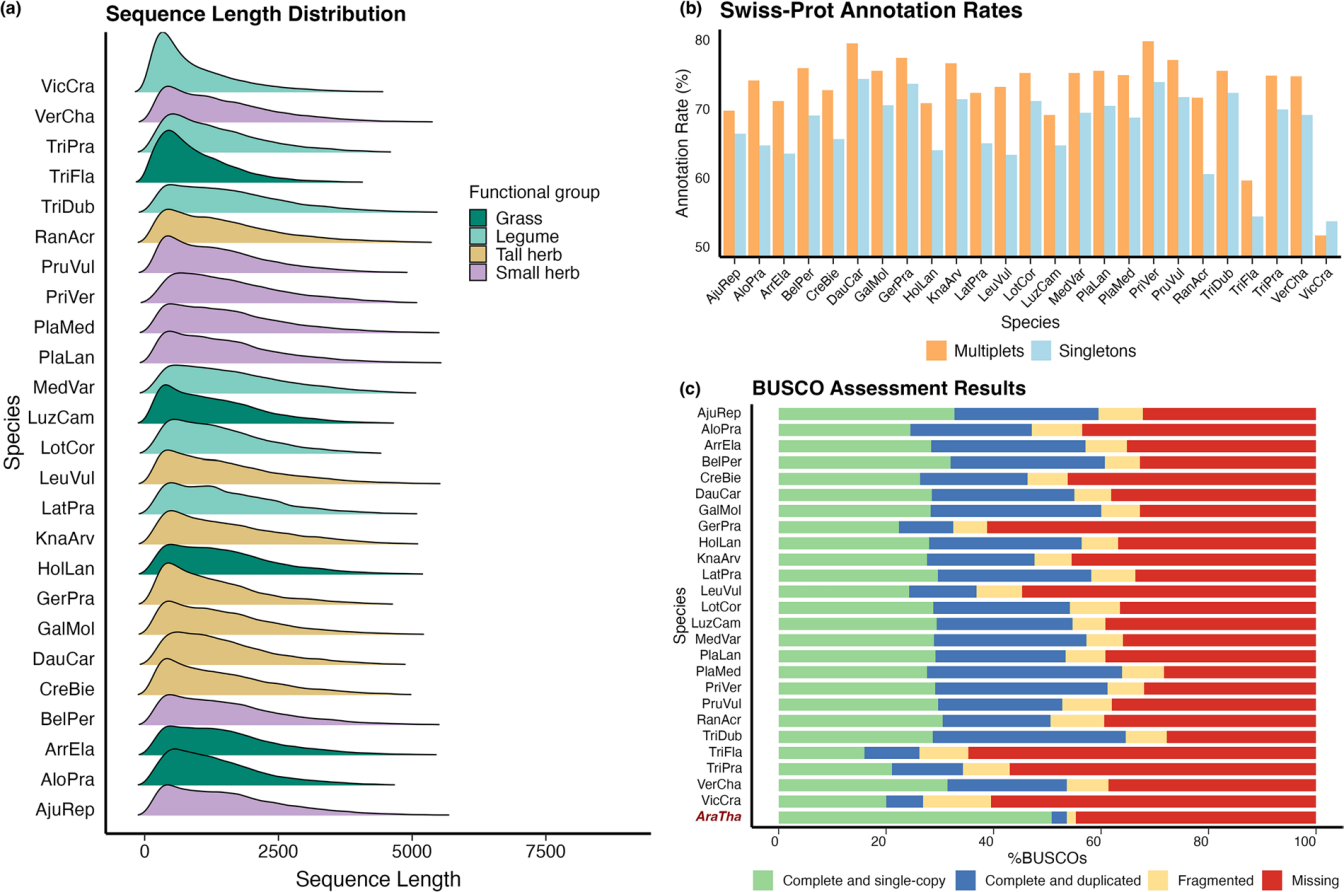
Analysis and Annotation of Iso-seq Transcripts. (**a**) Length distribution of Iso-seq transcripts. (**b**) Proportions of Iso-seq transcripts annotated by Swiss-Prot (Evalue ≤1 × 10^−10^). (**c**) BUSCO annotation results. “*AraTha*” represents *Arabidopsis thaliana* and other species are abbreviated as shown in Table 1.

Raw sequencing data were processed using the PacBio Iso-Seq pipeline. Circular Consensus Sequences (CCS) were generated from subreads using the ***ccs*** tool (version 6.4.0) (https://ccs.how) with default parameters. This routine identifies high-fidelity full-length reads based on multiple passes for each molecule. On average, 191,105 CCS reads were obtained per species (Table 2). These CCS reads were further processed with ***lima*** (version 2.9.0, using the*–isoseq* option) (https://lima.how) to remove sequencing adapters and barcodes. Poly(A) tails and artificial concatemers were removed using the ***isoseq*** tool (version 4.0.0) (https://isoseq.how), yielding Full-Length Non-Chimeric (FLNC) reads. The FLNC reads were then clustered using ***isoseq cluster*** to generate polished transcripts, with the*–singletons* option enabled to retain singleton transcripts. On average, 49,180 FLNC reads were generated per species (Table 2), with read lengths ranging from 51 to 8,685 bp (Fig. 1).

### Functional annotation

To annotate the full-length transcripts, the FLNC reads were aligned against the Swiss-Prot protein database (release 2024-07-24) using ***BLASTx*** (version 2.14.0)^39^. The BLASTx search was conducted with an E-value cutoff of 1 × 10^−10^, allowing for an accurate identification of homologous proteins. This annotation provided functional insights into the transcripts detected in the transcriptomes. On average, 68.6% of the transcripts per species were successfully annotated, which corresponded to 9,128 unique proteins on average (see Table 2 for each species). Multiplet transcripts (those supported by at least two FLNC reads) consistently showed higher annotation rates compared to singleton transcripts (supported by a single FLNC read), though the difference was less than 10% (Fig. 1).

We used ***BUSCO*** (Benchmarking Universal Single-Copy Orthologs)^40^ to further assess the completeness of the assembled transcripts. For comparison, published leaf transcript data from Arabidopsis thaliana were included. Reads (GEO accession: GSM6589889)^41^ were mapped to the *A. thaliana* coding sequences (CDS) using Salmon (v1.10.2). The CDS reference was obtained from the TAIR database (Araport11 CDS, 2022-09-14)^42^. This comparative analysis enables the assessment of transcriptome quality and completeness in grassland plants relative to a well-characterized model species (Fig. 1).

### Open reading frames (ORF) and non-coding RNA (ncRNAs) prediction

**Fig. 2:**
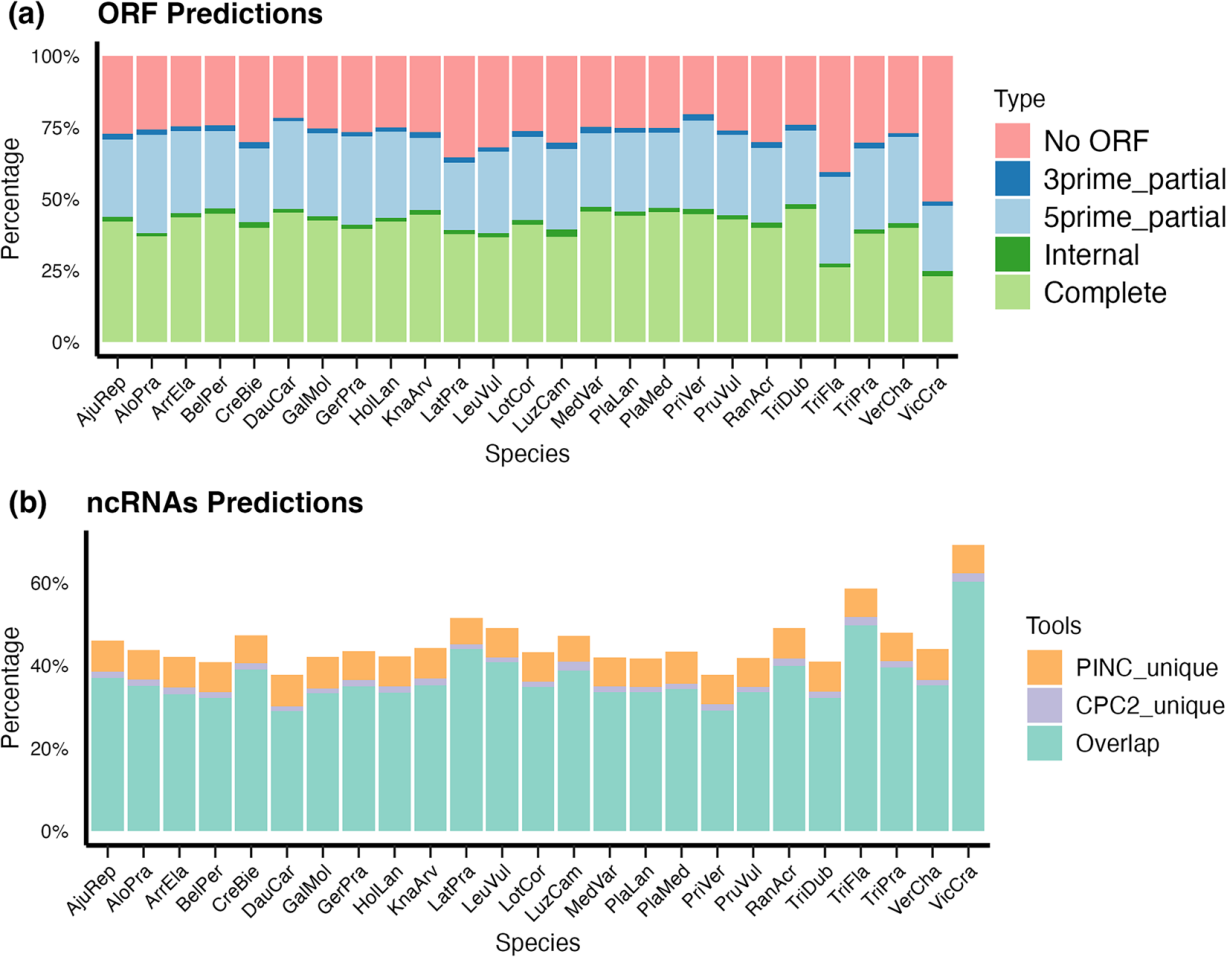
Structural Elements Identified in Iso-seq Transcripts. (**a**) Predicted open reading frames (ORFs) across species. (**b**) Predicted non-coding RNAs (ncRNAs) across species. Species are abbreviated as shown in Table 1.

ORF prediction was performed using ***TransDecoder*** (v5.7.1) (https://github.com/TransDecoder/TransDecoder/wiki). We identified 40.3% complete ORFs, encompassing both a start and stop codon, and 31.4% partial ORFs (Fig. 2). Non-coding RNAs (ncRNAs) were predicted using the ***CPC2*** (v 0.1)^43^ and ***PINC***^44^. The ncRNAs predicted by both tools showed significant overlap. On average, the overlap predicted by the two tools indicates that 36.8% of transcripts are non-coding RNAs (Fig. 2). Among transcripts for which TransDecoder did not identify an ORF, 96.7% (ranging from 95% to 98%) were also predicted as ncRNAs by CPC2 and PINC.

### Repetitive elements prediction

Repetitive elements within the transcriptomes were identified using ***RepeatMasker***^45^ (version 4.1.7-p1) (Table 3) with the Viridiplantae library, which was combined from RepBase^46^ (20181026 release) and Dfam^47^ (3.1 release). On average, 5.08% of the bases across the 25 species were flagged as repetitive elements. Of these, 1.96% were classified as retroelements, and 1.03% were classified as small RNAs. To further investigate the distribution of the repetitive elements, we categorized transcripts based on the proportion of their bases identified as repetitive elements (0–0.1%, 0.1–10%, 10–50%, and 50–100%). This analysis was performed separately for coding and noncoding sequences across 25 species (Table 3).

**Table 3:** Proportion of transcripts with varying percentages of repetitive element content.

Species	Non-coding*				Coding**			
	[0, 0.1%]	(0.1%, 10%]	(10%, 50%]	(50%, 100%]	[0, 0.1%]	(0.1%, 10%]	(10%, 50%]	(50%, 100%]
*Ajuga reptans*	64.22	20.36	12.42	3	59.45	32.03	6.68	1.84
*Alopecurus pratensis*	74.78	11.87	10.52	2.82	57.12	32.52	9.19	1.17
*Arrhenatherum elatius*	70.32	14.43	11.9	3.35	49.91	37.61	10.69	1.79
*Bellis perennis*	69.55	17.65	9.68	3.12	55.35	36.98	6.9	0.77
*Crepis biennis*	67.65	18.55	11.22	2.57	54.29	35.97	8.41	1.33
*Daucus carota*	78.09	12.76	7.31	1.84	65.95	28.27	5.45	0.33
*Galium mollugo agg*.	77	13.33	7.4	2.27	63.76	30.12	5.59	0.54
*Geranium pratense*	75.17	11	9.63	4.2	64.58	28.19	6.89	0.34
*Holcus lanatus*	73.05	14.17	9.91	2.86	51.97	37.59	9.19	1.25
*Knautia arvensis*	64.85	18.32	13.21	3.63	55.88	33.95	7.85	2.32
*Lathyrus pratensis*	57.34	12.92	26.93	2.8	57.13	33.65	8.04	1.18
*Leucanthemum vulgare agg*.	73.87	16.31	7.52	2.31	63.12	29.25	6.61	1.02
*Lotus corniculatus*	68.09	16.61	11.48	3.82	57.06	33.42	8.33	1.18
*Luzula campestris*	61.93	18.05	17.18	2.84	50.54	41.21	7.63	0.62
*Medicago x varia*	59.35	20.08	15.34	5.23	51.14	38.73	8.47	1.66
*Plantago lanceolata*	73.41	15.72	8.61	2.27	61.22	32.08	6.06	0.64
*Plantago media*	72.09	17.59	8.21	2.11	61.64	31.4	5.95	1.01
*Primula veris*	66.87	22.09	8.66	2.38	54.21	39.27	5.94	0.58
*Prunella vulgaris*	71.09	15.46	10.47	2.98	57.95	34.55	6.91	0.59
*Ranunculus acris*	69.22	19.26	9.34	2.19	60.17	31.04	7.3	1.49
*Trifolium dubium*	66.52	17.66	12.85	2.97	51.78	39.9	7.23	1.09
*Trifolium pratense*	65.75	16.48	13.28	4.49	56.76	34.45	7.52	1.27
*Trisetum flavescens*	77.38	8.06	10.98	3.58	57.37	29.08	11.77	1.77
*Veronica chamaedrys*	81.34	10.04	6.37	2.26	67.94	25.75	5.32	0.99
*Vicia cracca*	74.76	10.12	11.14	3.98	64.36	26.63	7.25	1.76
**Average**	**70.15**	**15.56**	**11.26**	**3.03**	**58.03**	**33.35**	**7.49**	**1.14**

Transcripts are categorized into four groups based on the proportion of bases flagged as repetitive elements (0–0.1%, 0.1–10%, 10–50%, and 50–100%).

*Noncoding RNA were the overlap identified by CAPT and CPC2; **Coding RNA are the rest transcripts. Numbers are percentages of bases.

## Data Records

All RNA-seq raw reads are deposited on NCBI Sequence Read Archive (SRA) under accession number SRR31054526, SRR31054525, SRR31054524, SRR31054523, SRR31054522, SRR31054521, SRR31054520, SRR31054519, SRR31054518, SRR31054517, SRR31054516, SRR31054515, SRR31054514, SRR31054513, SRR31054512, SRR31054511, SRR31054510, SRR31054509, SRR31054502, SRR31054503, SRR31054504, SRR31054505, SRR31054506, SRR31054507, SRR31054508^48–72^. The final processed transcriptomes can be found on Dryad (http://datadryad.org/stash/share/U68M8mKIX8XzgA-MJDuNW5WvWFlnqc8wnwTPGtZrqQM). Moreover, the results of Swissprot annotation^73^, ORF prediction^74^, ncRNA prediction^75^, repetition element prediction^76^ were deposited in figshare.

## Technical Validation

Chromosome-level genome assemblies are available for four out of the 25 species (Table 4). To assess the quality of the Iso-Seq assemblies, we mapped species-specific Iso-Seq data to these four genomes using ***MiniMap2***^77^ (version 2.26-r1175). Since long-read sequencing pipelines often generates redundant isoform models due to sequencing artifacts and alternative splicing, we employed ***TAMACollapse***^78^ (version 2023_03_28) to collapse redundant isoforms. Over 70% of the transcripts were mapped to unique genomic positions, resulting in an average of about two transcripts per gene for each species (Table 4).

**Table 4:** Mapping statistics on published genomes.

Species	Assembly level	Genome size	Total isoforms	Unique Mapped	Mapped Proportion	Transcripts	Genes
*Trifolium dubium*	Chromosome	679.1 Mb	62,797	54,880	87.39%	44,908	20,070
*Primula veris*	Chromosome	436.2 Mb	47,363	41,175	86.93%	30,891	12,509
*Daucus carota*	Chromosome	440.7 Mb	39,936	31,432	78.71%	25,630	11,361
*Trifolium pratense*	Chromosome	413.6 Mb	26,684	19,959	74.80%	16,192	8,884

## Data Availability

ccs: version 6.4.0, default parameters lima: version 2.9.0, main parameters: --isoseq --num-threads 4 isoseq: version 2.9.0, main parameters: refine --num-threads 4 --require-polya isoseq: version 2.9.0, main parameters: cluster --verbose --num-threads 4 --singletons BLASTx: version 2.14.0, main parameters: -evalue 1e-10 -num_threads 16 -max_target_seqs1 BUSCO: version 5.4.7, main parameters: --offline -l embryophyta_odb10 -m tran -c 4 -f Salmon: version 1.10.2, default parameters TransDecoder: version 5.7.1, default parameters CPC2: version 0.1, default parameters PINC: default parameters (executed using a Docker image) RepeatMasker: version 4.1.7-p, default parameters MiniMap2: version 2.26-r1175, main parameters: -t 10 -ax splice:hq -uf -G 6000 TAMACollapse: version 2023_03_28, main parameters: -x no_cap -d merge_dup
